# Apple EIN3 BINDING F-box 1 inhibits the activity of three apple EIN3-like transcription factors

**DOI:** 10.1093/aobpla/pls034

**Published:** 2012-11-10

**Authors:** Emma J. Tacken, Hilary S. Ireland, Yen-Yi Wang, Jo Putterill, Robert J. Schaffer

**Affiliations:** 1 The New Zealand Institute of Plant and Food Research, Private Bag 92169, Auckland 1142, New Zealand; 2 School of Biological Sciences, University of Auckland, Private Bag 92019, Auckland 1142, New Zealand

## Abstract

**Background and aims:**

Fruit ripening in *Malus*× *domestica* (apple) is controlled by ethylene. Work in model species has shown that following the detection of ethylene, the ETHYLENE INSENSITIVE 3 (EIN3) transcription factor is stabilized, leading to an increase in transcript accumulation of ethylene-responsive genes, such as *POLYGALACTURONASE1* (*PG1*). In the absence of ethylene, the EIN3 BINDING F-box (EBF) proteins rapidly degrade EIN3 via the ubiquitination/SCF (Skp, Cullin, F-Box) proteasome pathway. In this study, we aim to identify and characterize the apple *EBF* genes, and test their activity against apple EIN3-like proteins (EILs).

**Methodology:**

The apple genome sequence was mined for *EBF*-like genes. The expression of *EBF*-like genes was measured during fruit development. Using a transient assay in *Nicotiana benthamiana* leaves, the activity of three apple EILs was tested against the *PG1* promoter, with and without ethylene and EBF1.

**Principal results:**

Four *EBF*-like genes in apple were identified and grouped into two sub-clades. Sub-clade I genes had constant expression over fruit development while sub-clade II genes increased in expression at ripening. EBF1 was shown to reduce the transactivation of the apple *PG1* promoter by the EIL1, EIL2 and EIL3 transcription factors in the presence of ethylene.

**Conclusions:**

The apple *EBF1* gene identified here is likely to be a functionally conserved *EBF* orthologue, modulating EIL activity in apples. The activity of EBF1 suggests that it is not specific to a single EIL, instead acting as a global regulator of apple EIL transcription factors.

## Abstract (precis)

Mining the apple genome identified four *EIN3 BINDING F-BOX* genes, two developmentally regulated and two constitutively expressed. One (*EBF1*) was found to reduce the activity of three apple *EIN3-like* genes

## Introduction

Ethylene is involved in a wide range of developmental processes in plants including seed germination, cell elongation, sex determination, fruit ripening, senescence and leaf abscission, as well as biotic and abiotic stress responses (Abeles and Biles 1991; Barry and Giovannoni 2007; Lin *et al.* 2009). The ethylene response pathway can be briefly summarized as follows: the pathway is thought to be predominantly linear, consisting of ethylene receptors, which in the absence of ethylene constitutively repress the activity of the MAP kinase CONSTITUTIVE TRIPLE RESPONSE1 (CTR1); this modulates the activity of ETHYLENE INSENSITIVE 2 (EIN2), which results in the destabilization of the ETHYLENE INSENSITIVE 3 (EIN3) transcription factors. In the presence of ethylene this pathway is repressed and EIN3 is stabilized, initiating a transcriptional cascade leading to an ethylene response (Chen *et al.* 2005).

In the absence of ethylene, EIN3 is short-lived with a half-life of <30 min due to rapid degradation through the ubiquitin/Skp, Cullin, F-Box degradation pathway (Guo and Ecker 2003; Yanagisawa *et al.* 2003). In *Arabidopsis*, two redundant nuclear localized F-box proteins, EIN3-BINDING F BOX PROTEIN1 (AtEBF1) and AtEBF2, were shown to target EIN3 and a functional homologue EIN3-like 1 (EIL1) for degradation (Guo and Ecker 2003; Potuschak *et al.* 2003; Binder *et al.* 2007). Loss-of-function *ebf1 ebf2* double mutants had high levels of EIN3 protein and consequently exhibited a constitutive ethylene response (Chao *et al.* 1997; Potuschak *et al.* 2003). While EBF1 and EBF2 in *Arabidopsis* are constitutively expressed over plant development, they both show an increase in expression with exogenously added ethylene, and over-expression of *EBF1* resulted in reduced EIN3 levels leading to an ethylene-insensitive phenotype. These results suggest that the *EBF*-like genes are controlled, at least in part, at the transcription level (Potuschak *et al.* 2003).

In tomato, two *EBF*-like genes have also been identified: *EBF1* and *EBF2* (Yang *et al.* 2010). Consistent with the results in *Arabidopsis*, silencing of either gene resulted in plants that were indistinguishable from controls, indicating that they are functionally redundant. The results suggested a feedback mechanism whereby suppression of one *EBF* gene resulted in an increase in transcription of the second. As in *Arabidopsis*, a constitutive ethylene response phenotype was observed when both *EBF1* and *EBF2* were silenced in tomato, including accelerated fruit ripening (Yang *et al.* 2010). However, unlike *Arabidopsis*, the expression of tomato *EBF1* and *EBF2* was not constitutive, with a transient decrease in expression at the onset of ripening (mature green), and consistent with *Arabidopsis* both showed an increase of expression with ethylene and a decrease with auxin (Yang *et al.* 2010). Tomato *EBF1* appeared to be less affected at the transcriptional level, while *EBF2* appeared to be more transcriptionally variable (Yang *et al.* 2010).

In the fleshy fruiting apple, ethylene plays a key role in the control of fruit ripening. The importance of ethylene in apple fruit ripening was confirmed with the suppression of the ripening-associated ethylene biosynthesis gene *ACC OXIDASE 1* (*ACO1*). In these apples, no ripening-associated flesh softening or aroma volatiles are produced (Schaffer *et al.* 2007; Johnston *et al.* 2009). Owing to consumer requirements to maintain a firm texture, many commercial apples have been selected for low ripening-related ethylene. This has been achieved in part through the selection of lines with disrupted ethylene biosynthetic gene *ACC SYNTHASE* (*ACS*), leading to longer storage capacity and slower softening (Harada *et al.* 1997; Costa *et al.* 2005; Wiersma *et al.* 2007; Wang *et al.* 2009). Owing to the importance of ethylene in fruit ripening, much of the molecular biology research conducted in apple has been focused on ethylene biosynthesis and response. One of the earliest genes cloned from apple was the *ETHYLENE RESPONSE 1* (*ETR1*)-like receptor (Lee *et al.* 1998), along with the ethylene biosynthetic gene *ACO1* (Lay-Yee and Knighton 1995). Subsequent work identified four other receptor-like genes, a *CTR1*-like gene, an *EIN2-*like gene (Wiersma *et al.* 2007) and three *EIN3*-like genes (Tacken *et al.* 2010). With the release of the complete apple genome sequence (Velasco *et al.* 2010), there is now a growing literature studying whole gene families (Devoghalaere *et al.* 2012), which has led to the identification of three further receptor genes in apple (Ireland *et al.* 2012).

While five *EIN3*-like genes have been identified in *Arabidopsis*, ethylene signal transduction occurs predominantly through the action of two of them, EIN3 and EIL1. Originally identified through an ethylene-insensitive phenotype, it was proposed that EIN3 acted by binding and activating the promoters of the AP2/ERF class of transcription factors (Solano *et al.* 1998). Since this study, it has been shown that EIN3-like transcription factors are likely to be involved directly in the activation of a suite of ethylene biosynthesis and response genes (Huang *et al.* 2010; Tacken *et al.* 2010; Yin *et al.* 2010), and transient assays suggest that EIL2 and EIL3 in apple may be involved in the up-regulation of key apple ripening genes such as the cell wall hydrolase *endo-POLYGALACTURONASE 1* (*PG1*) (Tacken *et al.* 2010).

Owing to the importance of the *EBF* class of genes as key controllers of the ethylene signal transduction pathway, this study used the apple genome sequence to identify *EBF*-like genes. One *EBF*-like gene (*EBF1*) was cloned and tested for the ability to inhibit the activity of three EILs in a *Nicotiana benthamiana* transient assay.

## Methods

### Identification of the apple EBF genes and generation of a phylogeny

EBF-like genes were mined from the predicted peptide models from the apple genome using BLASTP. To verify the DNA sequence of the selected gene models, the DNA sequence from each *EBF-*like gene was compared with expressed sequence tag (EST) sequences. Predicted amino acid sequences were aligned in Geneious Pro™ version 4.8.4 (Biomatters, Auckland, New Zealand) (Drummond *et al.* 2011). Phylogenetic trees were created in Geneious Pro™ using the PHYML substitution method (Guindon and Gascuel 2003) with the JTT model (Jones *et al.* 1992). A total of 1000 replicates of each tree were used to generate bootstrap data. EBF sequences from other species used to construct the phylogenetic tree were: *Fragaria vesca* FvEBF1 (strawberry gene model 1520754), FvEBF2 (gene model 1540140) (**www.rosaceae.org**), the *Malus* gene models shown in Table 1 and EBF-like protein sequences drawn from published work (Yang *et al.* 2010); *Arabidopsis thaliana* AtEBF1 (NP_565597), AtEBF2 (NP_197917), AtFBL4 (NP_567467), AtTIR1 (NP_567135), AtZTL (NP_568855), *Brassica oleracea* BoF-box (ACB59221), OsF-box (BAD15849), *Populus trichocarpa* PtEBF3 (EEE92188), PtEBF4 (EEE92505), PtF-box (EEF03786), *Solanum lycopersicum* SlEBF1 (ACS44349) and SlEBF2 (ACS44350).

**Table 1:** Apple EBF-like genes.

Gene name	Gene model		Chromosome	Position (Mb)
*EBF1*	MDP0000239011	MDP0000314942	8	18.66
*EBF101*	MDP0000429728	MDP0000280142	15	5.98
*EBF2*	MDP0000230402		15	10.55
*EBF102*	MDP0000165656		2	3.02

### Quantification of gene expression

Gene expression levels from a fruit development cDNA series (Janssen *et al.* 2008) were determined via quantitative polymerase chain reaction (qPCR) using the Lightcycler480™ (Roche, Basel, Switzerland). Primers for *PG1*, *ACO1* and *EIL1-3* are as described in Tacken *et al*. (2010), and for *ACTIN* as described in Espley *et al*. (2007). Primers to measure the expression of each of the *EBF* genes were as follows: EBF1F, TCGCAAGAGGTCTCGCATCAGC; EBF1R, CCTCGCCTCCAGGAATCCGT; EBF101F, TTCCTGCTTGGGATTGAAAGATG; EBF101R, GCTCCAGTTGAGGGCAAAGC; EBF2F, AGGTTGTGCCCTCAGCTACATAATA; EBF2R, ACCAACGACACAACTGCTTTATCC; EBF102F, GCCCTCAGCTCCATAATGTAGACA; EBF102R, CCAACGCCATAACGACTTCATCT.

All reactions were carried out in quadruplicate using SYBR^®^ Green Master Mix (Roche) according to the manufacturer's instructions with *ACTIN* used as the reference gene, and the qPCR products sequenced to verify the amplification of the correct gene.

### Determination of activation using the dual luciferase transient assay system

Tobacco plants were grown in the greenhouse for 2 weeks under long-day conditions until at least two leaves had developed a surface area of at least 1.5 cm^2^. *Agrobacterium tumefaciens* GV3101 transformed with promoter fragments in the pGreenII 0800:Luc vector and the pSOUP helper plasmid (Hellens *et al.* 2000) and *Agrobacterium* containing the candidate *EIL*s or *EBF1* fused to the *CaMV35S* promoter in the pART7/27 transformation vector were suspended in 8 mL of infiltration buffer (Hellens *et al*. 2005) to obtain an optical density at 600 nm of 0.6 *Agrobacterium*. The leaves of young *N.benthamiana* plants were infiltrated with two aliquots of 500 μL of combined *PG1* promoter/*EIL*/*EBF1* at a ratio of 1 : 3.5 : 3.5. In the controls, *Agrobacterium* containing either the *EIN3*-like genes or *EBF1* was substituted for *Agrobacterium* containing an empty *CaMV35S* promoter construct (Voinnet *et al.* 2003; Hellens *et al.* 2005). Plants were grown for 3 days and then four independent leaf punches were assayed using a Berthold Orion Microplate Luminometer (Berthold, Bad Wilbad, Germany) according to the specifications for the dual luciferase assay (Hellens *et al.* 2005). Luminescence was calculated using Simplicity software, version 4.02 (Berthold). To minimize the effect of background activation levels, only readings with a Renilla value of >1000 were included in the analysis. These infiltrations were repeated three times and the averages of these experiments are given. Significant differences were calculated using analysis of variance.

## Results

### Identification of apple *EBF*-like genes

**Fig. 1:**
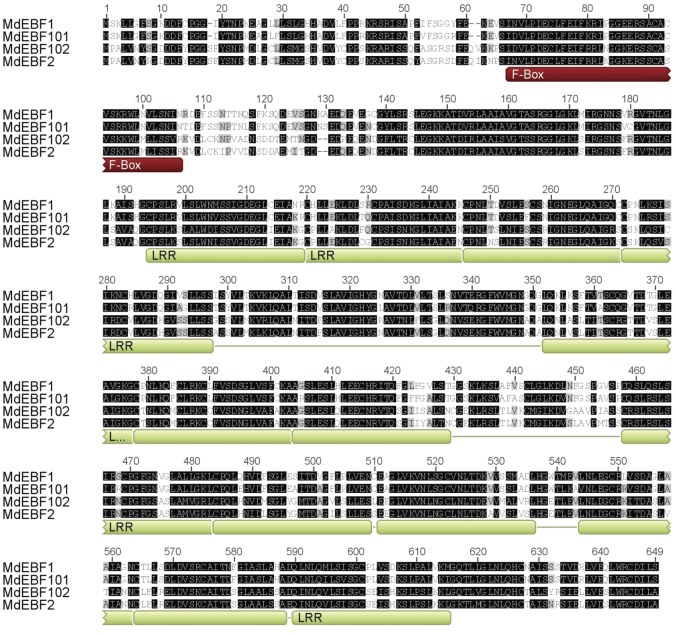
**Alignment of the EBF1 protein sequences.** The four predicted apple EBF proteins were aligned. The conserved F-box domain (red) and the 13 leucine-rich repeats (LRR—green) are shown underneath.

**Fig. 2:**
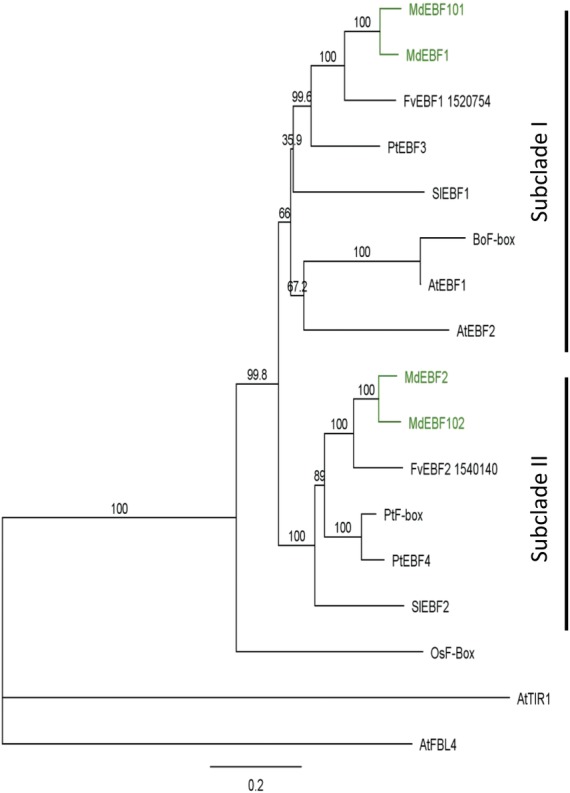
**Phylogenetic alignment of members of the EIN3 BINDING F-box (EBF) family proteins from different plant species.** A phylogenetic tree was generated using PHYML; values given are bootstrap percentages (1000 replicates). EBF-like proteins from apple (*Malus domestica—*Md), strawberry (*Fragaria vesca—*Fv), poplar (*Populus trichocarpa—*Pt), tomato (*Solanum lycopersicum—*Sl), *Brassica oleracea* (Bo), rice (*Oryza sativa—*Os) and *Arabidopsis thaliana* (At) were compared with AtFBL4 and AtTIR used as outgroups.

The protein sequences of *Arabidopsis* EBF1 and EBF2 were used to identify EBF-like genes within the predicted peptide models from the apple genome (Velasco *et al.* 2010) using BLASTP. Six gene models with a high BLAST score (*P* < e-150) were selected. The next highest apple model (MDP0000224875) had a considerably lower BLAST score (*P* < e-37) and only showed homology in the N-terminal F-box region, suggesting that this was unlikely to be within the EBF group of F-box proteins. When these proteins were aligned each was found to have the expected F-box region, and leucine-rich repeats were found in EBF-like genes (Fig. 1). Reciprocal BLASTP comparisons of the six apple peptide models with the *Arabidopsis* proteins selected EBF1 and EBF2 as the most similar *Arabidopsis* proteins. The six apple peptide models aligned to four unique chromosomal locations: two on chromosome 15, one on chromosome 2 and one on chromosome 8 (Table 1). Two of the chromosomal loci had two models each, suggesting that apple has four *EBF*-like genes. To test whether the gene models were correctly constructed, the DNA sequences of the four predicted protein sequences were compared with sequences from both an apple EST collection (Newcomb *et al*. 2006) and short read (100 bp) data from mRNA seq analysis from ripe ‘Royal Gala’ fruit (Schaffer *et al.* 2012). In two cases the predicted gene models differed from the EST sequences, firstly *EBF1* (with two gene models MDP0000314942 and a shorter model MDP0000239011) both extended 3′ beyond the region covered by ESTs. A single clone from a ‘Royal Gala’ cDNA library was fully sequenced, verifying that the gene was shorter in length than the gene models supplied (GenBank JX512439). When this new sequence was translated, the C-terminus was more consistent with the length of the *Arabidopsis* and tomato *EBF* genes. Secondly, the model for EBF2 (MDP0000230402) was 25 amino acids longer than the other EBF-like proteins at the N-terminus. Alignment of mRNA seq reads to the apple genome suggested that this model was incorrectly annotated at the 5′ end, with these new data the start codon was consistent with other EBF-like proteins [see Additional information: Supplemental Data 1]. Phylogenetic alignment was conducted with the four predicted apple EBF-like proteins, two genes selected in a similar way from the *Fragaria vesca* (strawberry) genome (Shulaev *et al.* 2011) and the EBF-like proteins from Yang *et al.* (2010). The phylogenetic alignment showed that the selected apple proteins fell into the same clade as the *Arabidopsis* (EBF1 and EBF2) and tomato (EBF1 and EBF2) proteins, suggesting that these were likely to be apple EBF orthologues (Fig. 2). The four apple proteins were separated into two proteins per sub-clade, with each sub-clade containing a single strawberry protein. This duplication was consistent with the ancient genome duplication event reported in apple (Velasco *et al.* 2010). The four selected apple *EBF*-like genes were assigned gene names as described in Devoghalaere *et al.* (2012). As both *Arabidopsis* EBF1 and EBF2 fell into sub-clade I containing tomato EBF1, the apple genes were named by the closest tomato genes, with strawberry EBF1 and the apple homeologues EBF1 and EBF101 grouping with the tomato EBF1 gene, and strawberry EBF2 and apple homeologues EBF2 and EBF102 grouping with tomato EBF2 in sub-clade II (Fig. 2).

### Analysis of EBF1 expression

**Fig. 3:**
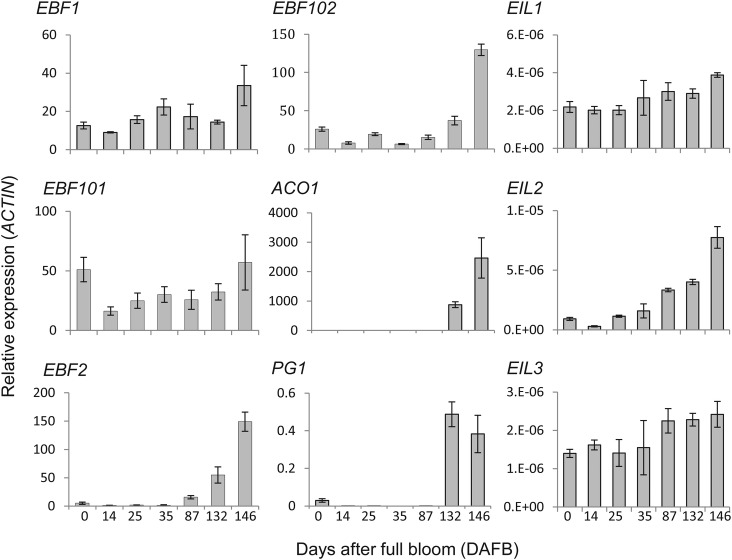
**Expression patterns of *EBF*-like genes over apple fruit development, compared with other ethylene-related and ripening genes.** Expression of *EBF1*, *EBF2*, *EBF3* and *EBF4* was measured in cDNA derived from fruit tissue over the course of apple fruit development by qPCR. Expression levels are shown relative to the *ACTIN* gene. Expression levels of *ACO1*, *PG1* and *EIL1*-3 are reported in Tacken *et al*. (2010).

The expression of the EBF genes during apple fruit development was compared with that of known ethylene biosynthesis genes (*ACO1*), potential EBF-like targets *EIL1*, *EIL2* and *EIL3* (Tacken *et al.* 2010) and the cell wall modifying gene *PG1* (Fig. 3). Expression of *EBF1* and *EBF101* was similar to that of *EIL1* and *EIL3*, and did not change significantly over the course of fruit development or at the onset of fruit ripening at 132 days after full bloom (DAFB), though a slight increase in expression was observed at 146 DAFB (Fig. 3). The expression of EBF2 and EBF102 was low early in fruit development, increasing as the fruit matured and ripened. This expression was more consistent with that of ethylene-responsive genes such as *ACO1* and *PG1*, which had a significant increase in expression at the onset of fruit ripening (data from Tacken *et al*. 2010).

### Functional analysis of EBF1 in a transient assay

**Fig. 4:**
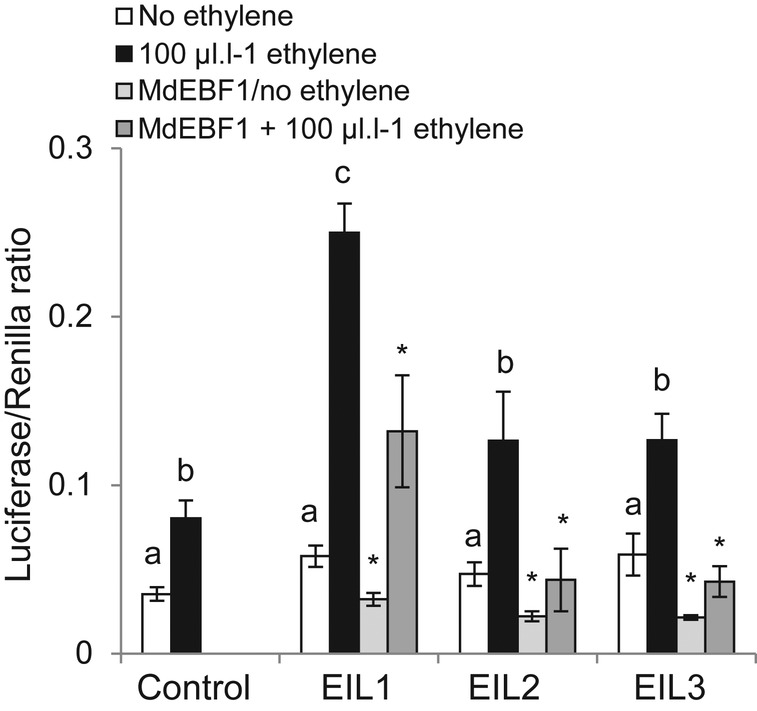
**The transactivation of the apple PG1 promoter by EIL1 to EIL3 with and without EBF1.** A dual luciferase transient assay system was used to examine the activity of EBF1 on the transactivation of the *PG1* promoter by EIL1, EIL2 and EIL3 with and without exogenous ethylene (100 μL L^-1^). Transactivation was measured as a ratio of luminescence from the luciferase activity compared with an infiltration control measured by Renilla activity. Controls are the *PG1* promoter and an empty vector control; error bars represent the S.E.M. (*n* = 4). Letters depict bars that are significantly different with a *P* value <0.05, and an asterisk indicates significant levels of inactivation (*P* < 0.05).

It has previously been shown that a 2.6-kb apple *PG1* promoter fused to the *LUCIFERASE* gene can be trans-activated when injected into a *N. benthamiana* leaf in the presence of exogenous ethylene (Tacken *et al.* 2010). When the *EIL2* and *EIL3* transcription factors, driven by a *CaMV35S* promoter, were co-injected with the *PG1* promoter in the presence of ethylene, an increased transactivation of the *PG1* promoter occurred, especially with *EIL2* (Tacken *et al.* 2010). To test whether the EBF1 protein can destabilize the apple EIL proteins and thus block their transactivation of *PG1*, the *EIL2* and *EIL3* constructs as well as a construct containing *EIL1* were co-infiltrated with the *PG1* promoter, with and without EBF1. Each assay was performed either in the presence or absence of ethylene. In this study, apple EIL1 trans-activated the PG1 promoter in the presence of ethylene to a much higher level than EIL2 and EIL3 (Fig. 4). When co-infiltrated with the *EBF1* gene, the levels of trans-activation were greatly reduced with all three apple EILs, consistent with the activity of an EBF-like F-box protein. Interestingly, a level of inhibition by the EBF1 was also observed in non-ethylene-treated leaves. This suggests that the act of infiltrating *Agrobacterium* into the *N. benthamiana* leaves may elicit an ethylene-induced defence response in the leaves, which by itself can trans-activate the *PG1* promoter (Fig. 4).

## Discussion

A rapidly growing number of plant genomes have now been sequenced, giving researchers a valuable insight into these organisms beyond the traditional model species. While these genomes allow researchers to look at features that are unique to different and often commercially important plant species, it is important to translate knowledge gained from model systems to these species of interest. In this study, we build on the growing literature of ethylene-related genes in apple (Lee *et al.* 1998; Wiersma *et al.* 2007; Tacken *et al.* 2010; Ireland *et al.* 2012) by the characterization of the *EBF*-like genes. Interestingly, in apple there are four *EBF*-like genes, consistent with the genome duplication, while the closely related Rosaceae species strawberry has two. In the model species tomato and *Arabidopsis*, the *EBF* family is encoded redundantly by at least two genes. In *Arabidopsis* the two *EBF* genes fall into sub-clade I, while tomato has one gene in each sub-clade (Fig. 2).

In *Arabidopsis, EBF1* and *EBF2* mRNA is constitutively expressed (Guo and Ecker 2003; Potuschak *et al.* 2003) and has been shown to be targeted for degradation by EIN5 (Olmedo *et al.* 2006), suggesting that mRNA levels are actively regulated. In tomato, *EBF1* is constitutively expressed with *EBF2* showing considerable changes in expression over development and in different treatments (Yang *et al.* 2010). From this observation it was suggested that as *EBF1* had a more consistent level of expression, it was providing the steady-state level of EBF, and fluctuations of *EBF2* allowed the plant to respond to the environment. In apples, the two classes of *EBF*-like genes appear to follow the same pattern with sub-clade I genes (*EBF1* and *EBF101*) showing little variation in expression, while the sub-clade II genes (*EBF2* and *EBF102*) both increase as the fruit begin to ripen. Although the sub-clade I tomato gene *EBF1* had a more consistent level of expression, it did have lower expression in mature green fruit. This was not observed in the expression pattern of *EBF1* in apples, though as this drop was transitory in tomato, there is a possibility that a similar drop in apple would be missed in a less detailed time series experiment (Fig. 3).

In this study, three apple EIN3-like genes were tested in a transient assay for activity against the *PG1* promoter. All three apple EILs had reduced activity against the *PG1* promoter, in the presence of EBF1, showing that the apple EBF1 was not specific to a single EIL. The non-specific nature of the EBFs is consistent with the *Arabidopsis* EBF1 and EBF2, where both interact with EIN3 and EIL1, again suggesting a lack of specificity in these F-box proteins to individual EIL proteins.

## Conclusions and forward look

An F-box gene *EBF1* was identified in apple, the predicted protein product of which clustered with EBF-like protein*s* involved in the ethylene response in other plant species. *EBF1* negatively regulated activation of *PG1* by the apple *EILs*, consistent with the degradation of EIN3 by EBF1 and EBF2 observed in *Arabidopsis* and tomato. These results also suggest that apple EBF1 acts as a functional EBF upon multiple members of the EIL family of transcription factors. This work suggests that the *EBF*-like genes in apple are likely to play a crucial role in the control of ethylene-related fruit ripening.

## Additional information

The following additional information is available in the online version of this article –Text files of apple *EBF* DNA sequences and predicted proteins.

## Accession numbers

Apple EBF1 GenBank accession no. JX512439.

## Sources of funding

This work was funded by The Agriculture and Marketing Research and Development Trust of New Zealand (AgMardt), The University of Auckland, New Zealand and the Foundation of Science and Research Technology (FRST) contract C06X0705; Pipfruit, a juicy future.

## Contributions by the authors

The project was conceived, executed and the first draft written by E.J.T. Sequencing, cloning and expression analysis were undertaken by E.J.T. H.S.I. and Y.-Y.W. This work was part of E.J.T.'s PhD project funded by AgMardt PhD scholarship (NZ), supervised by and edited by R.J.S. and J.P.

## Appendix

The complete references with the full list of authors for Shulaev *et al.* (2011) and Velasco *et al.* (2010) are as follows:

**Shulaev V, Sargent DJ, Crowhurst RN, Mockler TC, Folkerts O, Delcher AL, Jaiswal P, Mockaitis K, Liston A, Mane SP, Burns P, Davis TM, Slovin JP, Bassil N, Hellens RP, Evans C, Harkins T, Kodira C, Desany B, Crasta OR, Jensen RV, Allan AC, Michael TP, Setubal JC, Celton JM, Rees DJ, Williams KP, Holt SH, Ruiz Rojas JJ, Chatterjee M, Liu B, Silva H, Meisel L, Adato A, Filichkin SA, Troggio M, Viola R, Ashman TL, Wang H, Dharmawardhana P, Elser J, Raja R, Priest HD, Bryant DW, Jr, Fox SE, Givan SA, Wilhelm LJ, Naithani S, Christoffels A, Salama DY, Carter J, Lopez Girona E, Zdepski A, Wang W, Kerstetter RA, Schwab W, Korban SS, Davik J, Monfort A, Denoyes-Rothan B, Arus P, Mittler R, Flinn B, Aharoni A, Bennetzen JL, Salzberg SL, Dickerman AW, Velasco R, Borodovsky M, Veilleux RE, Folta KM. 2011.** The genome of woodland strawberry (*Fragaria vesca*). *Nature Genetics* **43**: 109–116.

**Velasco R, Zharkikh A, Affourtit J, Dhingra A, Cestaro A, Kalyanaraman A, Fontana P, Bhatnagar SK, Troggio M, Pruss D, Salvi S, Pindo M, Baldi P, Castelletti S, Cavaiuolo M, Coppola G, Costa F, Cova V, Dal Ri A, Goremykin V, Komjanc M, Longhi S, Magnago P, Malacarne G, Malnoy M, Micheletti D, Moretto M, Perazzolli M, Si-Ammour A, Vezzulli S, Zini E, Eldredge G, Fitzgerald LM, Gutin N, Lanchbury J, Macalma T, Mitchell JT, Reid J, Wardell B, Kodira C, Chen Z, Desany B, Niazi F, Palmer M, Koepke T, Jiwan D, Schaeffer S, Krishnan V, Wu C, Chu VT, King ST, Vick J, Tao Q, Mraz A, Stormo A, Stormo K, Bogden R, Ederle D, Stella A, Vecchietti A, Kater MM, Masiero S, Lasserre P, Lespinasse Y, Allan AC, Bus V, Chagne D, Crowhurst RN, Gleave AP, Lavezzo E, Fawcett JA, Proost S, Rouze P, Sterck L, Toppo S, Lazzari B, Hellens RP, Durel C-E, Gutin A, Bumgarner RE, Gardiner SE, Skolnick M, Egholm M, Van de Peer Y, Salamini F, Viola R. 2010.** The genome of the domesticated apple (*Malus x domestica* Borkh.). *Nature Genetics* **42**: 833–839.
